# Auditory Brainstem Response Data Preprocessing Method for the Automatic Classification of Hearing Loss Patients

**DOI:** 10.3390/diagnostics13233538

**Published:** 2023-11-27

**Authors:** Jun Ma, Jae-Hyun Seo, Il Joon Moon, Moo Kyun Park, Jong Bin Lee, Hantai Kim, Joong Ho Ahn, Jeong Hun Jang, Jong Dae Lee, Seong Jun Choi, Min Hong

**Affiliations:** 1 Department of Software Convergence, Soonchunhyang University, Asan 31538, Republic of Korea; ringring369@gmail.com; 2 Department of Otorhinolaryngology-Head and Neck Surgery, Seoul St. Mary’s Hospital, College of Medicine, The Catholic University of Korea, Seoul 06591, Republic of Korea; revivalseo@catholic.ac.kr; 3 Department of Otorhinolaryngology-Head and Neck Surgery, Sungkyunkwan University School of Medicine, Samsung Medical Center, Seoul 06351, Republic of Korea; mooniljoon@gmail.com; 4 Department of Otorhinolaryngology-Head and Neck Surgery, Seoul National University Hospital, Seoul National University College of Medicine, Seoul 03080, Republic of Korea; aseptic@snu.ac.kr; 5 Department of Otorhinolaryngology-Head and Neck Surgery, Konyang University College of Medicine, Daejeon 35365, Republic of Korea; rogue25@kyuh.ac.kr (J.B.L.); noto.hantai@gmail.com (H.K.); 6 Department of Otorhinolaryngology-Head and Neck Surgery, Asan Medical Center, University of Ulsan College of Medicine, Seoul 05505, Republic of Korea; ucanhear@gmail.com; 7 Department of Otolaryngology, Ajou University School of Medicine, Suwon 16499, Republic of Korea; jhj@ajou.ac.kr; 8 Department of Otorhinolaryngology-Head and Neck Surgery, Soonchunhyang University College of Medicine, Bucheon Hospital, Bucheon 14584, Republic of Korea; ljdent@schmc.ac.kr; 9 Department of Otorhinolaryngology-Head and Neck Surgery, College of Medicine, Soonchunhyang University, Cheonan Hospital, Cheonan 31151, Republic of Korea; akas9238@hanmail.net; 10 Department of Computer Software Engineering, Soonchunhyang University, Asan 31538, Republic of Korea

**Keywords:** deep learning, VGG, ABR, image processing, hearing loss

## Abstract

Auditory brainstem response (ABR) is the response of the brain stem through the auditory nerve. The ABR test is a method of testing for loss of hearing through electrical signals. Basically, the test is conducted on patients such as the elderly, the disabled, and infants who have difficulty in communication. This test has the advantage of being able to determine the presence or absence of objective hearing loss by brain stem reactions only, without any communication. This paper proposes the image preprocessing process required to construct an efficient graph image data set for deep learning models using auditory brainstem response data. To improve the performance of the deep learning model, we standardized the ABR image data measured on various devices with different forms. In addition, we applied the VGG16 model, a CNN-based deep learning network model developed by a research team at the University of Oxford, using preprocessed ABR data to classify the presence or absence of hearing loss and analyzed the accuracy of the proposed method. This experimental test was performed using 10,000 preprocessed data, and the model was tested with various weights to verify classification learning. Based on the learning results, we believe it is possible to help set the criteria for preprocessing and the learning process in medical graph data, including ABR graph data.

## 1. Introduction

Hearing loss refers to a condition in which the ability to hear or understand sounds is more difficult than for people with normal hearing due to abnormalities in the inner, outer, or vestibulocochlear nerves. These symptoms may be temporary or permanent and may affect one or both ears. Auditory brainstem response (ABR) refers to an electrical response that occurs when sound is recognized in the brainstem, which is the path through which sound (audio) reaches the auditory cortex of the brain as a neuroelectric signal of the cochlear tube. The diagnostic process used to assess the hearing threshold by measuring the waveform of the corresponding electrical response is usually referred to as an ABR test.

The ABR test, generally performed to evaluate hearing loss, is a non-invasive test that is unaffected by sleep or anesthesia. In particular, the ABR test is a means of objectively measuring the hearing of newborns, infants, and patients with congenital disabilities who have difficulty accurately measuring hearing level, and due to the objectivity of non-invasive testing, plays an important role in tracking the hearing of children at risk of late-onset or progressive hearing loss, such as those affected by congenital CMV infection [1,2]. The hearing threshold of the pure tone audiometry test is the smallest sound that the subject can hear in each frequency band at (125 to 8000) Hz. However, the ABR test uses clicks, tone pips, or tone bursts about 0.8 ms apart to audibly stimulate the brain and records changes in brain waves due to energy induced by stimuli modulation during sound transmission. An auditory stimulation ranging from approximately 10 to 100 dBHL is given, and waves l to V are detected. In normal adults, a waveform that responds to the stimulus appears within approximately 10 ms of starting a click sound stimulus. In the case of using a click sound, the auditory threshold is 35 dBnHL or more, and in the case of using a tone pip and tone burst, the auditory threshold is 40 dBnHL or more, referred to as hearing loss [3,4,5,6,7]. Among them, in audiological investigations, recordings are made at different levels of stimulus intensity until a response (wave V) is no longer observed, and this is taken as an estimation of the hearing threshold [8].

The ABR test is a process by which small electrodes are attached to the forehead and behind the ears to detect electrical activity in the auditory nerve and brainstem in response to sound. The patient hears a series of clicking sounds through earphones inserted into the ears, and the brain waves, which are the brain’s response to the sounds, are detected and automatically recorded on a computer. Therefore, the ABR test can be measured objectively, compared to test methods based on the patient’s subjective response, such as pure tone audiometry. The audiologist judges the hearing threshold of the ABR test with waveform 5, which is detected between 6 and 8 ms, which is the most reliable among waveforms 1 to 5 from each sound stimulus (dB). The ABR test is completed after measuring the EEG for each (30 to 90) dB sound stimulus [9,10].

**Figure 1:**
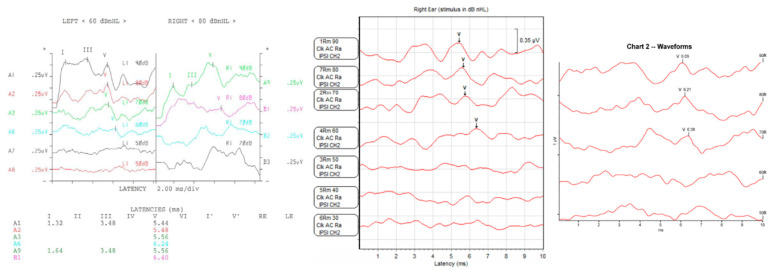
ABR test results of different forms from various manufacturers.

In general, the ABR test results can determine hearing thresholds, the presence of pseudohypacusis, and retro-cochlear hearing loss [11]. Despite its many advantages, the ABR test has several limitations. The biggest limitation is that there is a high possibility that an error can occur depending on the experience and skill level of the audiologist in the hearing threshold or the hearing result value due to the subjective judgment of the audiologist. In addition, the ABR threshold corresponds reasonably well with the average subjective hearing threshold across the frequency range from 2 kHz up to 4 kHz and does not reflect hearing loss in the low-frequency region [12,13]. As shown in Figure 1, the ABR test results are displayed on the screen and stored in various forms because each hospital uses measuring equipment from different manufacturers. Because of the different measuring devices, audiologists need experience in using the specific devices, and it is difficult to collect ABR data in the same format for deep learning.

These problems also occur in the proposed hearing loss classification AI model using ABR data. When the ABR result data of each manufacturer are directly applied to the AI model and trained without refining, low-accuracy prediction results are obtained. In this paper, to develop an AI learning model that automatically classifies hearing loss, the preprocessing process for efficiently learning ABR data is proposed. After applying the proposed preprocessing process, we trained the VGG16 model for data and the classification test for hearing loss patients.

The ABR data in this paper were obtained by gathering data from Soonchunhyang University Cheonan Hospital, Asan Medical Center, Samsung Medical Center, Ajou University Medical Center, Seoul National University Hospital, Severance Hospital, Seoul St. Mary’s Hospital, and Konyang University Hospital. The ABR data of 5000 people with normal hearing and 5000 patients with hearing loss were collected and preprocessed. Using the collected data, a deep learning model was applied to analyze the classification performance and accuracy of the deep learning model. This study was reviewed and approved by the Soonchunhyang University Cheonan Hospital’s Investigational Review Board (IRB number: 2021-06-040). We classified ABR image data into hearing loss and normal hearing based on the ABR hearing threshold of 40 dB. The main contribution of this paper is that the proposed method is designed to automatically distinguish between hearing loss patients and normal hearing people using the VGG16 model. The proposed method is expected to be clinically usable with an accuracy of approximately 85%.

## 2. Materials and Methods

### 2.1. Medical Data for Deep Learning

Medical data is the most actively researched in the field of AI, especially in deep learning technology, and is one of the fields in which many automatic diagnosis systems are currently being developed. Recently, as various medical data have been digitized and a large amount of high-quality medical information has been generated, research on computer-based analysis and automatic diagnosis has been actively conducted [14,15,16,17]. In addition, research to apply traditional machine learning to artificial intelligence technologies is also being conducted. However, despite active research on image diagnosis through machine learning, the results have not been good. The developed algorithm had limitations for actual clinical application due to poor medical accuracy and the lack of robustness of various data. Since algorithms in classical image processing techniques or machine learning methods are based on the developer’s intuition or experience, it is difficult to consider all the various situations, shapes, and transformations of data. Therefore, it is not easy to model complex and abstract transformations beyond the developer’s knowledge. This is considered a limitation of machine learning methods in medical imaging and remains at the research level without leading to actual clinical application or commercialization.

With the advent of deep learning technology, this perception and situation have considerably changed. Deep learning is a concept designed based on an artificial neural network (ANN) that showed limitations due to the initial overfitting problem, but Jeffrey Hinton presented the concept of prior learning in 2006 and the concept of dropout in 2012, and it has been proven that it can overcome the existing problems [14,18,19,20]. In particular, among various deep learning fields, the convolutional neural network (CNN) performs highly in image recognition. Research has been conducted based on CNN in medical imaging fields, including various fields of vision [21,22,23,24]. As a representative example, preoperative medical image analysis can be used to determine the accuracy of the surgical site and the severity of the condition. An objective analysis of traumatic lesions [25] or prescription of optimal nutrients using the patient’s unique vital data [26,27] are utilized using AI.

### 2.2. Auditory Brainstem Responses

**Figure 2:**
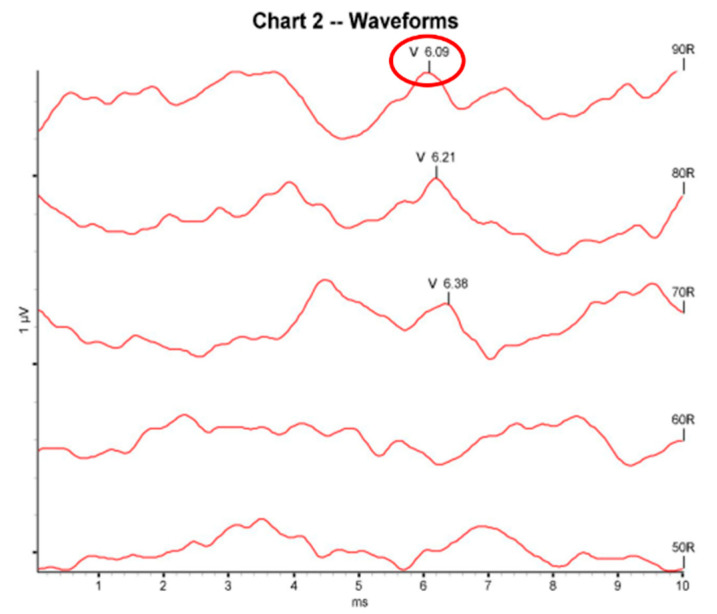
V waveform detection in an ABR result graph.

By attaching electrodes, the auditory brainstem response (ABR) test records electrical changes occurring in auditory conduction across the auditory nerve and brainstem after sound stimulation through the ear canal. Unlike pure tone audiometry (PTA), the ABR test is a non-invasive test that can objectively evaluate hearing threshold, is not affected by anesthesia or sleep, and is widely used in otolaryngology [28,29]. As shown in Figure 2, the ABR test determines whether the most reliable V waveform, related to hearing, is detected among the (I–V) waveforms transmitted from the brainstem. Based on dBHL, ISO 1964 [30], it is classified as normal if the V waveform is detected below 25 dBHL, mild hearing loss if the V waveform is detected in the (26 and 40) dBHL range, moderate hearing loss if the V waveform is detected in the (41 and 55) dBHL range, moderate–severe hearing loss if the V waveform is detected in the (56−70) dBHL range, and severe hearing loss if it is detected above 71 dBHL [31,32].

### 2.3. Data Processing and Cleansing

Hearing test results are stored in a medical information system, such as an electronic medical record (EMR), at each hospital. Patients’ personal information is removed manually by using data labelers. Since ABR data is stored in graph form from ABR devices used in each hospital, they should be collected and preprocessed (as shown in Table 1). Accuracy of the data can be increased by deep learning via unifying the different data formats through data preprocessing, and improving the data quality.

### 2.4. ABR Data Preprocessing Process

#### Data Normalization and Preprocessing

**Figure 3:**
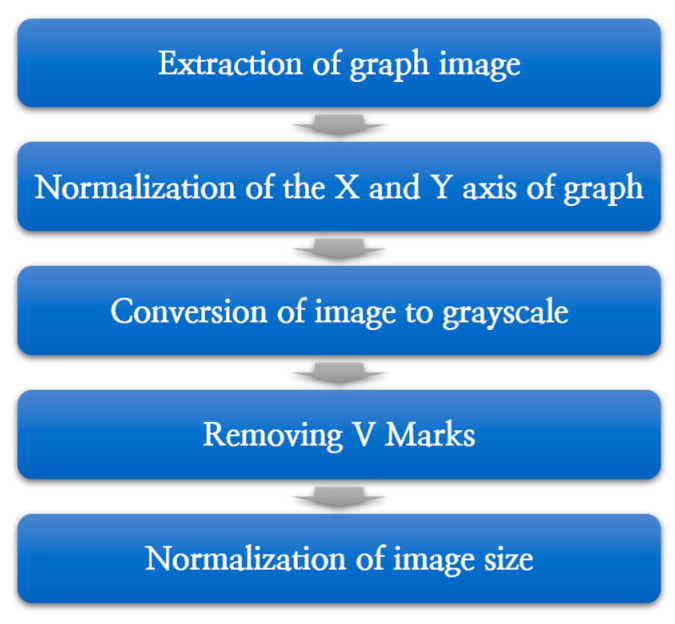
The proposed data preprocessing process.

Figure 3 is a flowchart of the preprocessing process performed to normalize the ABR data acquired from each hospital, and a detailed explanation of the proposed data preprocessing process follows.

1. Extraction of graph image

**Figure 4:**
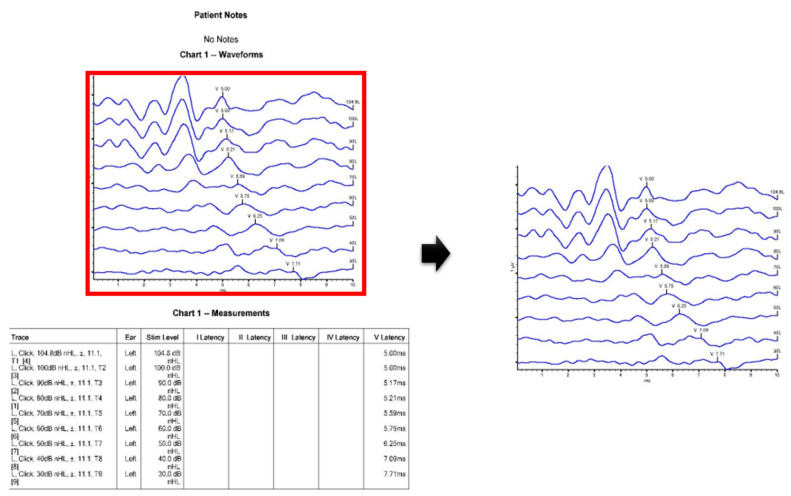
Extraction of graph image.

Figure 4 shows the process of extracting only the graph part necessary for image learning from the raw ABR data. In the collected ABR data, graph and table image data are stored together. We extracted only graph images to automatically determine whether there was hearing loss using graph images.

2. Normalization of the *X* and *Y* axes of the graph

**Figure 5:**
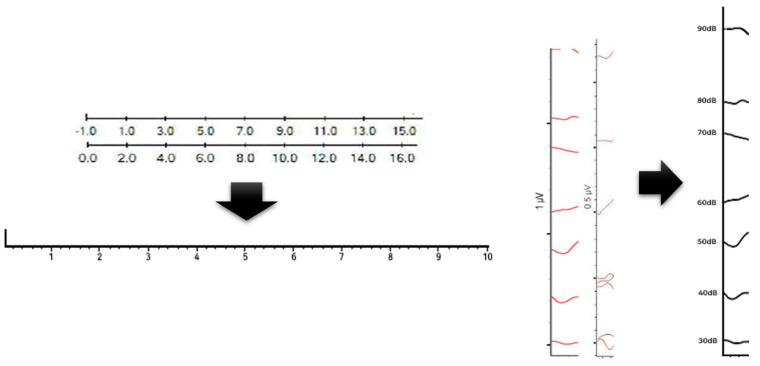
Normalization of the *X* and *Y* axes in a graph image.

Figure 5 shows the before and after normalization of the X and Y-axes for the ABR result data obtained from each hospital ABR device. Since the zero point standard is 0 or — value, and the scale standards of the axes are different from each other, which affects data learning and analysis, we proceeded to standardize the axes in the same format.

3. Conversion of image to gray-scale

**Figure 6:**
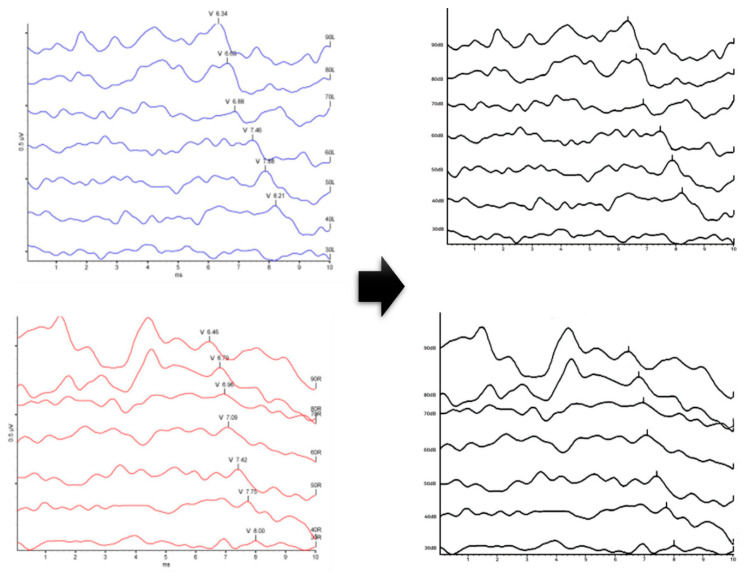
Image conversion to gray-scale.

Since the ABR test basically measures both ears, the ABR device is displayed in a separate color to distinguish the left ear from the right ear. The ABR data in this paper is a model to verify the classification of simple hearing loss, and since there is no need to distinguish between left and right ears, the image data is unified in gray-scale, as shown in Figure 6 below:

4. Removing V Marks

**Figure 7:**
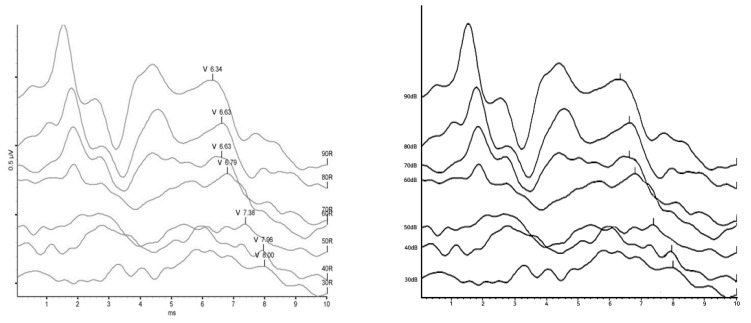
V Mark removal.

In the obtained ABR images, the V waveform notation method is not uniform, such as some graph images having scale marks and images with a separate mark (v) for feature point marks. Therefore, as shown in Figure 7, we deleted the separate V waveform mark for each graph and left only the vertical bar shape at the corresponding V position.

5. Normalization of image size

To smoothly process the deep learning for graph images, the image size was changed to 573 × 505 px for normalized and preprocessed images. Since *X* and *Y*-axis normalization and V-mark removal were already performed in the previous step, ABR image data loss for deep learning training was minimized.

In this paper, 10,000 ABR data from 8 different hospitals were collected, preprocessed, and normalized. In addition, a deep learning classification model was applied to the preprocessed ABR image data to classify patients with hearing loss automatically.

### 2.5. VGG 16 Model

**Figure 8:**
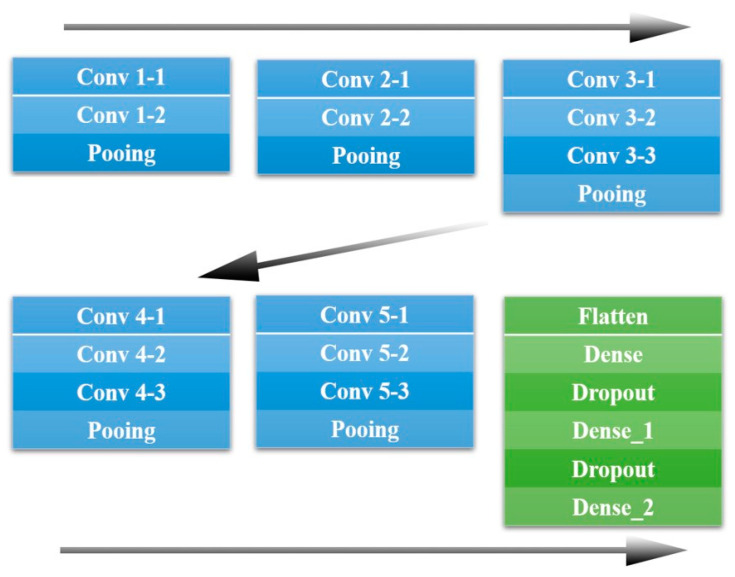
The VGG 16 Architecture.

VGG is a model proposed by the Visual Geometry Group of Oxford. There are two types of VGG structures: VGG16 and VGG19. VGG 19 has three convolution layers, one in front of the pooling layer in layers 3, 4, and 5, compared to VGG 16. For binary classification learning, the model in this paper adopted VGG 16 because too many convolution layers have a high probability of over-integration and may thus be less optimized [33,34,35]. Figure 8 shows the architecture of VGG16.

1. Input layer: Basically, an image of size 224 × 224 is delivered to the convolution layer;
2. Convolution layers: VGG16 has 13 convolution layers (conv 1–1~5–3). Each convolution layer consists of a small filter with a size of 3 × 3. Each filter is responsible for extracting features from the input image. After the convolutional layer, a rectified linear unit (ReLU) is used as the activation function;
3. Pooling Layers: After each convolution layer, the max pooling layer is applied. Maximum pooling is responsible for reducing space by extracting only the largest values from each area;
4. Dense Layers: These layers perform a final classification based on the features of the image. After each fully connected layer, the ReLU activation function is also used [36,37,38].

### 2.6. Structure of the Proposed VGG16 Model by Tuning Hyperparameter

**Figure 9:**
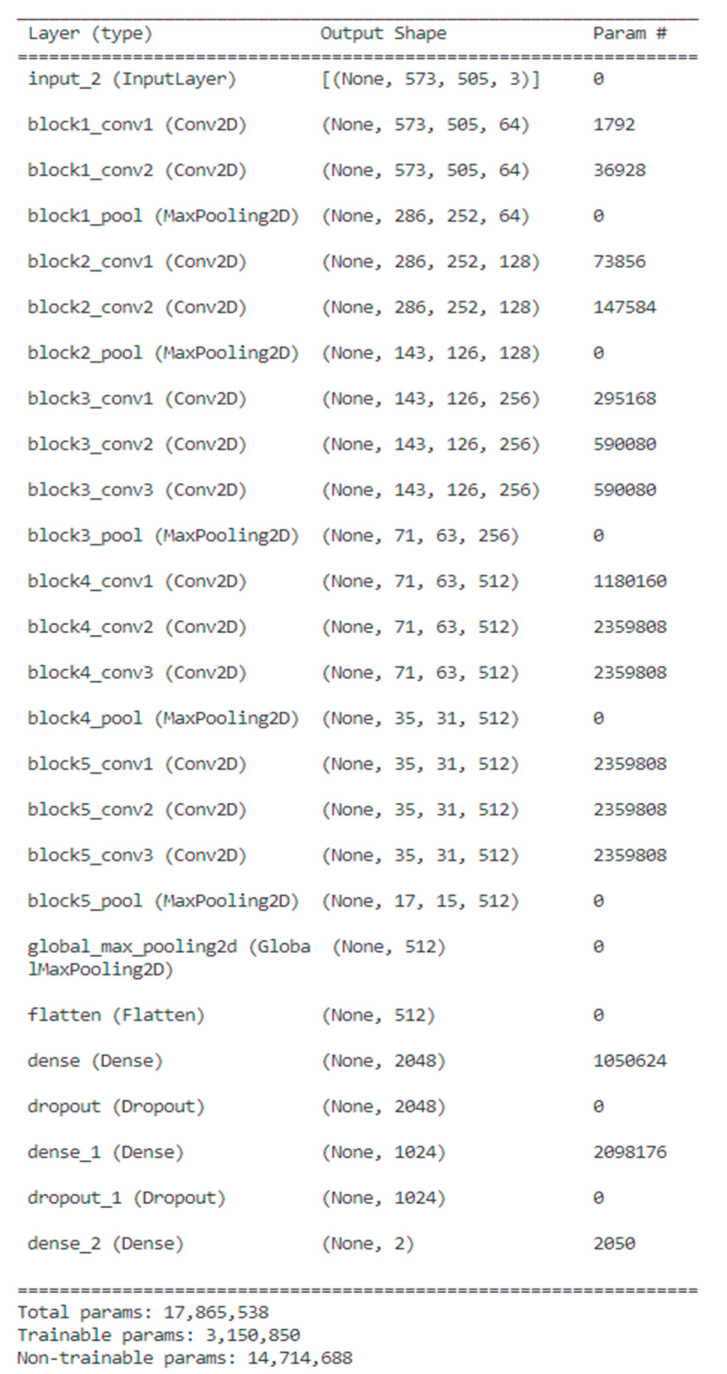
The structure of the VGG 16 model.

As shown in Figure 9, in the existing vgg16 model, we use 573 × 505 images rather than 224 × 224 images to perform object recognition learning by reducing the size to 286 × 252, 143 × 126, 71 × 63, 35 × 31, and 17 × 15 when passing through each convolutional layer, and the dence layer was also trained by adjusting the number of neurons to 2048, 1024, and 2 instead of 4096, 4096, and 2. A total of six cases were studied and tested, including two cases of batch size 8, 16 and three cases of epoch 50, 100, and 200 as hyperparameters of learning.

## 3. Results

In this paper, the VGG16 model for ABR data classification was used to classify hearing loss patients and people with normal hearing. To train the VGG16 model on the collected data, 4500 normal ABR images and 4500 hearing loss ABR images were used. To test the VGG16 model, 500 normal ABR images and 500 hearing loss ABR images were used. Table 2 shows the specifications of the deep learning machine that conducted learning and classification work in this study.

### VGG16 Model Learning and Classification Results

**Figure 10:**
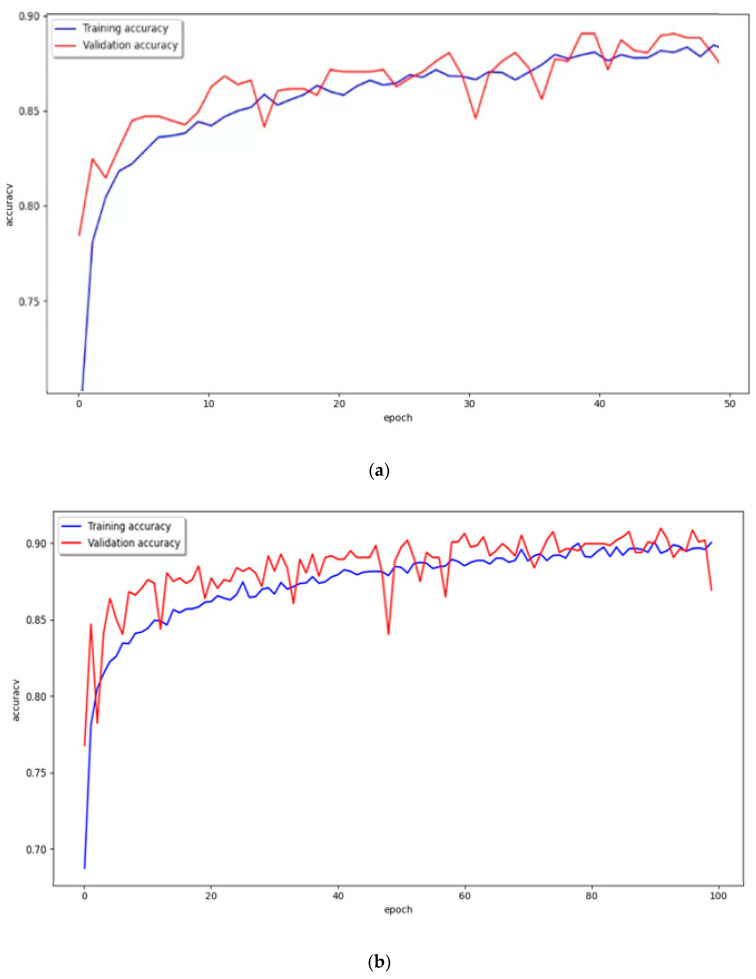

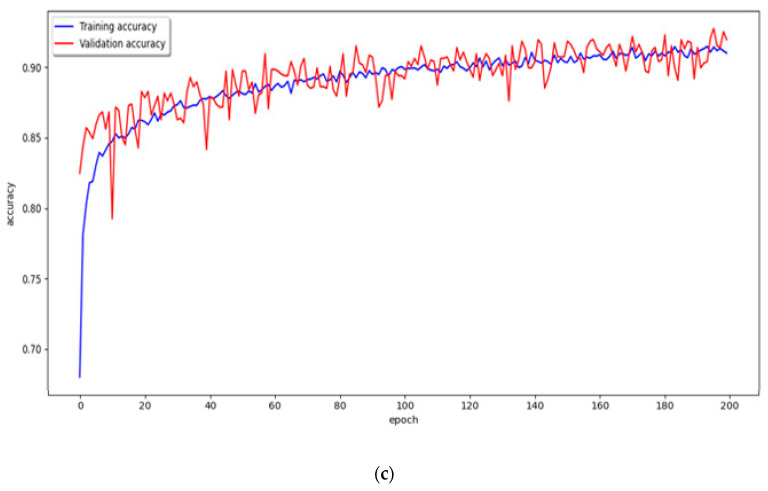
Graph of training and validation accuracy. (**a**) epoch 50, batch size 16; (**b**) epoch 100, batch size 16; (**c**) epoch 200, batch size 16. Blue line is training accuracy. Red line is validation accuracy.

**Figure 11:**
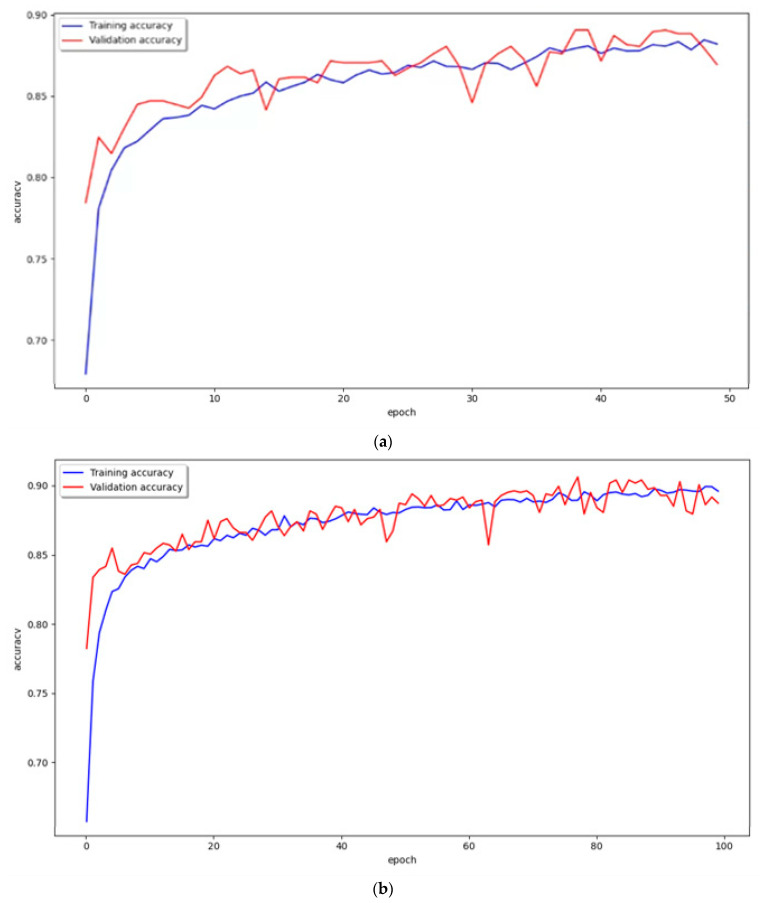

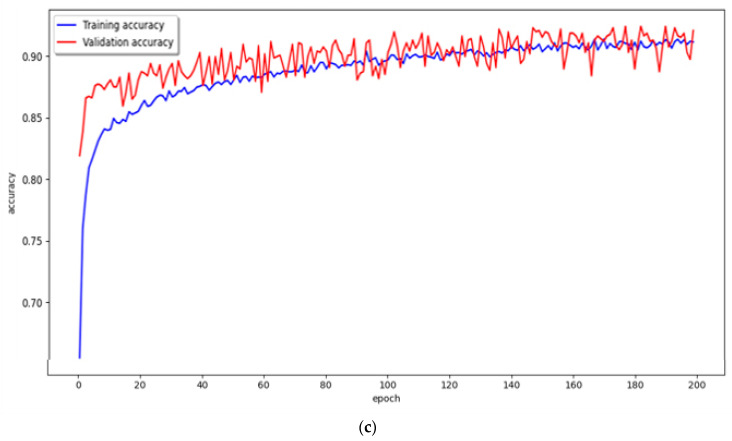
Graph of training and validation accuracy. (**a**) epoch 50, batch size 8; (**b**) epoch 100, batch size 8; (**c**) epoch 200, batch size 8. Blue line is training accuracy. Red line is validation accuracy.

To accurately evaluate the performance of the proposed VGG16 model, we tested and compared it under several conditions. In our experimental test of the proposed VGG16 model, the epochs were increased to (50, 100, and 200) for batch sizes (16 and 8). Therefore, six results were derived, and Figure 10 and Figure 11 and Table 3 show the performance results for the hearing loss patients classification after each learning.

## 4. Discussion

### Performance Evaluation of the VGG16 Model

Table 4 and Table 5 show the performance evaluation of the proposed VGG16 model. It shows the test result of 500 normal ABR images and 500 hearing loss ABR images analyzed by calculating the accuracy, specificity (TNR: true negative rate), sensitivity (TPR: true positive rate), FPR (false positive rate), FNR (false negative rate), precision, and F1 score. Prediction results for the hearing loss patient data consisted of four types: (1) True positive (TP) is when a normal patient is classified as normal. (2) False positive (FP) is when a patient with hearing loss is classified as normal. (3) True negative (TN) is a case in which a patient with hearing loss is classified as hearing loss. (4) False negative (FN) refers to cases of normal hearing loss patients classified as hearing loss patients. Equations (1)−(7) show the calculation equations for each performance evaluation.

Accuracy indicates the rate at which the proposed model accurately predicted whether the subject was a person with normal hearing (40 dB ≤ hearing threshold) or a patient with hearing loss (40 dB > hearing threshold). Sensitivity represents the rate at which people with normal hearing were properly classified as normal. The false positive rate (FPR) refers to the rate of actual hearing loss patients predicted to be people with normal hearing, while the false negative rate (FNR) refers to the rate of those who were predicted to be hearing loss patients, even though they were people with normal hearing. Precision represents the ratio of cases classified as people with normal hearing to actual people with normal hearing. The F1 score represents the harmonic average of precision and sensitivity. The higher the F1 score value, the more valid the value is. The primary purpose of this paper was to determine whether patients applying for disability-grade screening are people with normal hearing or hearing loss patients. Therefore, high sensitivity, precision, and low FNR, which do not classify people with normal hearing as hearing loss patients as much as possible, are judged as valid values while judging the accuracy value that reliably distinguishes people with normal hearing from hearing loss patients. In addition, the F1 score must also be high to verify the reliability of the corresponding value.

(1) $$\mathrm{Accuracy}=\frac{\mathrm{tp}\;+\;\mathrm{tn}}{\mathrm{tp}\;+\;\mathrm{tn}\;+\;\mathrm{fp}\;+\;\mathrm{fn}}$$

(2) $$\mathrm{Specificity}=\frac{\mathrm{tn}}{\mathrm{tn}\;+\;\mathrm{fp}}$$

(3) $$\mathrm{Sensitivity}=\frac{\mathrm{tp}}{\mathrm{tp}\;+\;\mathrm{fn}}$$

(4) $$\mathrm{FPR}=\frac{\mathrm{fp}}{\mathrm{fp}\;+\;\mathrm{tn}}$$

(5) $$\mathrm{FNR}=\frac{\mathrm{fn}}{\mathrm{fn}\;+\;\mathrm{tp}}$$

(6) $$\mathrm{Precision}=\frac{\mathrm{tp}}{\mathrm{tp}\;+\;\mathrm{fp}}$$

(7) $$F1\;\mathrm{score}=2*\frac{\mathrm{precision}\;\times \;\mathrm{sensitivity}}{\mathrm{precision}\;+\;\mathrm{sensitivity}}$$

The experimental results showed that when the epoch was set to 200 and the batch size was set to 8, it showed the best performance with accuracy results of 84.90% and an F1 score of 85.66%. The batch size refers to the number of data belonging to a small group when learning training image data once. A smaller number requires longer data learning time, but the results of the model used in this paper showed slightly better learning results. Epoch refers to the number of times the entire data passes through the neural network, and it simply refers to the number of learning times. Greater number epochs also require longer learning time, but a better learning rate is achieved when learning is carried out up to a certain upper limit.

To prove the effectiveness of the preprocessing performed in this paper, we conducted classification learning and testing for hearing loss patients and people with normal hearing using the VGG16 model. In addition to the best value of epoch 200, epochs 50 and 100 were also sufficiently effective for classification. Therefore, we confirmed that the preprocessing work proposed in this paper is effective in building a hearing loss patient classification model using ABR image data, and in future research, we plan to continue automating the preprocessing work. It is expected that this work will help to distinguish ABR data-based hearing loss between measurement technicians and otolaryngologists.

## 5. Conclusions

This paper introduced a preprocessing process to automatically classify patients with hearing loss using ABR graph image data using a deep learning model. A result of testing for automatic classification with the VGG16 model after preprocessing the ABR input image of this paper shows accuracy results of 84.90%, specificity of 79.60%, sensitivity of 90.20%, FPR of 20.40%, FNR of 9.80%, precision 81.56%, F1 score 85.66% under epoch 200, and batch size 8 were obtained. Among the 6 learning experimental tests, the FNR is low while the harmonic average F1 score is high, which explains that the rate of normal data classified as hearing loss patient data is low, while the reliability of the learning results is somewhat high. This is expected to help doctors conduct a second review after the first discrimination using the model when classifying the disabled with ABR graph data.

Currently, ABR data is measured by an audiologist using a specific ABR device, and then an otolaryngologist diagnoses hearing loss using these data. This study aims to analyze the ABR result automatically using AI technology for the primary classification of hearing loss, and then to proceed with the otolaryngologist’s secondary discrimination. This technique is expected to be of great help in improving the hearing loss diagnosis and analysis process. In a future study, we will present the automation of the ABR data preprocessing process and use the proposed model as new research. In another future study, we will apply an algorithm that automatically detects V waveforms to research a deep learning model to help audiologists evaluate ABR data.

## Figures and Tables

**Table 1:** Examples of representative differences in ABR graphs obtained from each hospital.

Difference	Example of ABR Data Image
Different graph colors for each device	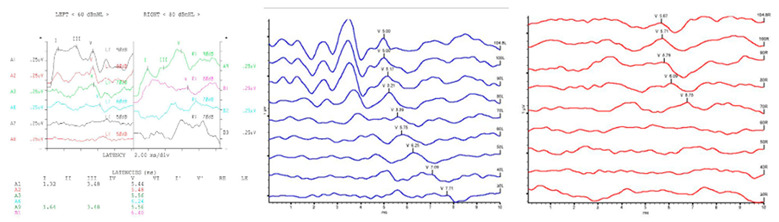
Inconsistency of *X*-axis starting point	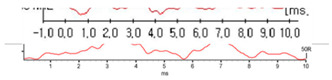
Inconsistency of V waveform mark	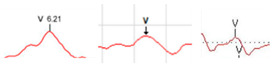
Inconsistency of the presence of graph grid	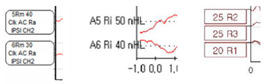

**Table 2:** Hardware specifications.

Part	Specification
OS	Windows 10 Pro
CPU	Intel Core i7−12700 2.10 GHz
GPU	NVIDIA GeForce RTX 4090 24 GB
RAM	Samsung 21400—32.0 GB*2
SSD	Samsung 970 1 TB

**Table 3:** Training accuracy of each test.

**Epoch/Batch Size**	**50/16**	**100/16**	**200/16**
Training accuracy	87.52%	88.20%	92.13%
**Epoch/Batch Size**	**50/8**	**100/8**	**200/8**
Training accuracy	88.12%	89.40%	91.96%

**Table 4:** Test result of batch size 16.

Epoch	50	100	200
Confusion matrix (tn;fp;fn;tp)	386;114;68;432	402;98;89;411	384;116;43;457
Test accuracy	81.80%	81.30%	84.10%
Specificity	77.20%	80.40%	76.80%
Sensitivity	86.40%	82.20%	91.40%
FPR	22.80%	19.60%	23.20%
FNR	13.60%	17.80%	8.60%
Precision	79.12%	80.75%	79.76%
F1 score	82.60%	81.47%	85.18%

**Table 5:** Test result of batch size 8.

Epoch	50	100	200
Confusion matrix (tn;fp;fn;tp)	405;95;106;394	430;70;95;405	398;102;49;451
Test accuracy	79.90%	83.50%	84.90%
Specificity	81.00%	86.00%	79.60%
Sensitivity	78.80%	81.00%	90.20%
FPR	19.00%	14.00%	20.40%
FNR	21.20%	19.00%	9.80%
Precision	80.57%	85.26%	81.56%
F1 score	79.68%	83.08%	85.66%

## Data Availability

No new data were created in this study.
